# First psychotic episode, related to COVID‐19 pandemic, in a patient with tuberous sclerosis complex

**DOI:** 10.1002/ccr3.4821

**Published:** 2021-09-21

**Authors:** Lina Brahmi, Hanen Ben Ammar, Safa Messaoud, Ghada Hamdi, Emira Khelifa, Leila Mnif

**Affiliations:** ^1^ Faculty of Medicine of Tunis Tunis El Manar University Tunis Tunisia; ^2^ Psychiatry Department “F” Razi Hospital Manouba Tunisia

**Keywords:** COVID‐19, Delusions, Psychotic disorder, Tuberous Sclerosis

## Abstract

Clinical symptoms of tuberous sclerosis may occur because of exposure to a stressful event like COVID‐19. During pandemics, specific considerations should be deserved to the mental state of people suffering from genetic diseases to prevent mental illness caused by a coronavirus.

## Graphical abstract

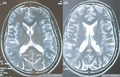

## 1 INTRODUCTION

The rapid spread of the SARS‐CoV‐2 pandemic worldwide and especially in Tunisia poses challenges to the management of both physical and mental health. This unexpected situation could predict multiple cases of psychosis. However, the psychological impact of the COVID‐19 pandemic on patients with genetic diseases such as tuberous sclerosis complex remains to be poorly understood. We report a case of a patient with tuberous sclerosis who presented to the emergency of a Tunisian psychiatric hospital with hallucinations, delusions, and disorganized behavior. He developed psychotic symptoms, which were directly triggered by stress derived from the COVID‐19 pandemic.

Coronavirus disease 2019 (COVID‐19) has so far affected over 188 million people worldwide and caused 4,05 million deaths.^1^ In the case of Tunisia, at the time of writing, the number of confirmed cases has now reached 510000, including nearly 16651 deaths.^1^ The rapid spread of the SARS‐CoV‐2 pandemic poses challenges to the management of both physical and mental health.^2^ In fact, it may cause psychological distress, especially in patients suffering from chronic diseases.

However, little information has been available about the psychological impact of COVID‐19 pandemic in patients with genetic diseases such as tuberous sclerosis complex (TSC).

TSC is an autosomal dominant and multisystem disorder.^3^ It has a birth incidence of approximately 1/6000.^4^ It is characterized by the triad of epilepsy, intellectual disability, and adenoma sebaceum.

Apart from the range of physical manifestations observed, many patients with TSC may develop neuropsychiatric manifestations at various levels with behavioral, intellectual, psychosocial components, defined as TSC‐associated neuropsychiatric disorders (TAND).^5^ This syndrome is unlikely to be well understood.

The prevalence of psychotic disorders is the same as that occurs in the general population (about 1%).^6^ Hallucinations and delusions are explained based on tubers impinging upon various limbic structures and causing dopaminergic dysfunction. They can also be associated with seizure disorders, particularly temporal lobe discharges.^7^

In this case report, we aim to present a patient with TSC diagnosed at an early age who first developed psychotic manifestations and disorganized behavior triggered by psychological distress related to the current coronavirus pandemic.

## 2 CASE REPORT

Mr. L.A, a 29‐year‐old male patient, from a rural place, presented to the emergency of Razi hospital, a Tunisian psychiatric hospital, with disorganized behavior, hallucinations, and delusions.

He had a recent history of impulsive and aggressive behaviors. Overt social isolation existed with under‐eating and insomnia. The onset was insidious with the context of the COVID‐19 pandemic. He was afraid of being infected, worried about the possibility of being a contagious asymptomatic carrier of coronavirus. He was isolated to avoid the spread of the disease. His family were struggled with financial loss due to quarantine.

Mr. L.A was diagnosed with TSC, epilepsy, and moderate intellectual disability at an early age without any psychotic manifestations. The prescribed medication for epilepsy was 600 mg of carbamazepine, 75mg of phenobarbital, and 1500 mg of valproate acid.

There was no family history of epilepsy, psychotic, or bipolar disorder.

Mental status examination revealed poor personal hygiene, lack of eye contact, flat affect, and a laconic speech. Attention, concentration, and short‐term memory were slightly impaired. There were delusions of reference, persecution, and demonic possession with an unshakable belief. There were verbal hallucinations in the form of female and male voices. He was convinced that everyone was talking about him behind his back. He had visual hallucinations of his pictures published on billboards around the world with a coronavirus sign above. Voices on television were talking about him and accused him to be responsible for this pandemic. Strangers claimed that he conspired with enemies to hurt people. Moreover, he believed that his skin lesion emitted waves rich in viruses to get people infected.

Because of his delusional ideas, he was no longer taking his antiepileptic drugs regularly, and he had multiple seizures with loss of consciousness during the last two weeks.

**FIGURE 1:**
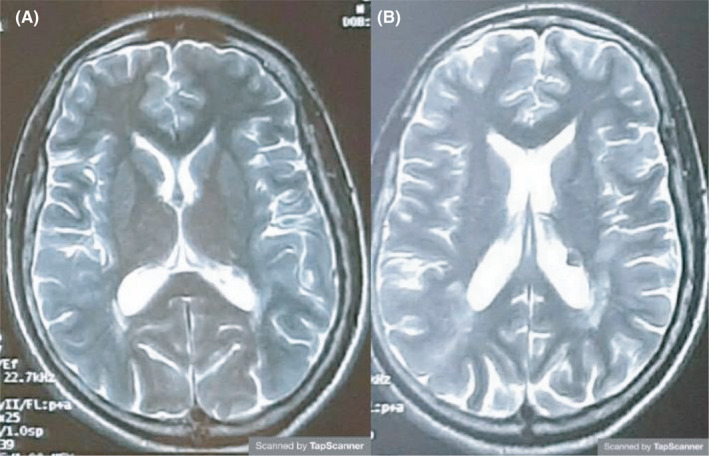
Previous (A) and current (B) magnetic resonance images of the brain. This figure shows cortical‐subcortical tuberous and subependymal nodules without any overt radiological aspect modification within the time course of the illness

Physical examination revealed hypopigmented macules in the forehead. An EEG revealed epileptic discharge foci in the temporal region. Previous and current magnetic resonance imaging of the brain demonstrated cortical‐subcortical tuberous and subependymal nodules compatible with TSC without any overt radiological aspect modification within the time course of the illness (Figure 1). The biological assessment was normal.

The patient received Olanzapine in the daily posology of 15 mg, in addition to an adjustment of antiepileptic treatment. The posology of phenobarbital was optimized until 150mg.

The carbamazepine and valproate acid blood levels were within the therapeutic range.

During treatment, Mr. L.A improved significantly with maintaining good seizure control, an improvement of mood, less intrusive thoughts, and the development of initial insight.

This case report was revised to comply with recommendations of the Case Report guidelines, and an informed consent publication was obtained from the patient and his father.

## 3 DISCUSSION

Patients with TSC may develop a variety of neuropsychiatric disorders called TAND. Neurodevelopmental disorders such as autism spectrum disorder (40–50%) and attention‐deficit hyperactivity disorder (30–50%) are the most common psychiatric diagnoses made.^8^ According to a recent study that enrolled 2216 participants with TSC from 170 sites across 31 countries to explore TAND syndrome, psychiatric disorders are underdiagnosed and potentially diagnosed late. Psychosis and hallucinations were nonfrequent manifestations but significantly higher in adults than children (10.3% vs 0.6%, *p*< 0.001).^9^

This case represents a form of TAND, where psychiatric symptoms, suggesting a psychotic disorder, occurred after many years of disease's evolution. Several etiological factors can be identified, and some hypotheses can be generated.

First, psychotic symptoms can be associated with epilepsy. It is known that early and multiple seizures are associated with a poor neurodevelopmental outcome, which may increase the risk of psychosis. Furthermore, psychosis may be precipitated by longer duration of epilepsy and higher frequency of seizures. In fact, frequent subclinical epileptic discharges and repeated altered consciousness may cause structural and functional damage, including neuronal changes, neurophysiological and blood flow abnormalities in the brain network across neocortical, limbic, and subcortical regions, which may result in pathological mental phenomena leading to developing psychotic symptoms.

As a matter of fact, this patient had EEG abnormality in the temporal lobe and associated epileptic seizures, which are considered as risk factors for psychosis. According to a recent meta‐analysis, the pooled prevalence of psychosis in epilepsy is 5.6% and 7% in temporal lobe epilepsy.^10^ Psychotic disorders identified in patients with epilepsy are commonly referred to in the medical literature as psychosis of epilepsy (POE).^11^ Epilepsy and POE have a complex and bidirectional relation. Not only are patients with epilepsy at increased risk of developing a psychotic disorder, but patients with a psychotic disorder are also at greater risk of developing epilepsy. POE generally exhibits mood disorder, anxiety, hallucinations, delusions, and disorders of consciousness. It can be classified according to the temporal relationship to the seizures as ictal, postictal, and interictal psychosis. The risk factors for postictal psychosis include temporal lobe epilepsy, earlier epilepsy onset, and impaired intellectual function, all of which were present in this case. However, clinical amelioration of seizures and psychotic symptoms, without a significant improvement in EEG patterns, suggests that psychiatric manifestations might be an independent component in this case.

Second, psychotic symptoms may also be caused by organic lesions due to TSC.^7^ The literature lacks knowledge about the pathogeny of these symptoms. Few cases reported the onset of psychiatric manifestations. According to Andrej N Ilanković et al, 5 patients with the average age of 35, 7 years, from different part of Yugoslavia, who were admitted between 2013 and 2016, developed paranoid psychotic episodes one year before being hospitalized.^12^ In fact, hallucinations and delusions are explained based on tubers impinging upon various limbic structures. Subependymal calcification, giant cell tumors, and retinal phakomas might be also the cause. However, there were not any overt modification in the cerebral imaging within the time course, which made this hypothesis less possible.

Third, the occurrence, in this patient, of a first psychotic episode triggered by a stressful event, comorbid with his organic pathology, remains highly probable. Indeed, all symptoms were reported to emerge in the month following the global emergence of the COVID‐19 pandemic. Although no explanation of the relationship between the pandemic and this case can be considered definitive, we suggest that a combination of social isolation, longer duration of quarantine reduced individual liberty, inadequate supplies, financial loss, fear of the infection itself, feeling insecure, a loss of control, and poor quality of sleep might have triggered an intense psychobiological stress reaction leading to the psychosis onset.^13^, ^14^

This acutely stressful scenario could play an important role in the emergence of new‐onset psychoses and might also be a major risk factor for clinical decompensation in individuals with previous chronic disorders who are considered as being vulnerable to psychiatric diseases.^15^, ^16^ As a matter of fact, a recent study made by María José Valdés‐Florida and al, including all the hospitalized patients with reactive psychoses during the first two weeks of quarantine, shows that relapses were directly triggered by stress derived from the COVID‐19 pandemic.^2^ Also, Zulkifli et al identified a case report from Malaysia, of an acute episode of psychosis seemingly precipitated by fear and distress associated with the COVID‐19 pandemic, in 31‐year‐old male patient without previous history of mental disorder, nor substance use.^12^ About medical care, there is not a specific treatment for psychosis seen in TSC.^17^ In literature, a case was reported responding well to risperidone without side effects.^17^ Our patient received olanzapine with marked improvement in psychotic symptoms.

## 4 CONCLUSION

The clinical symptoms of TAND may occur because of exposure to a stressful event like COVID‐19. These patients could be more substantially influenced by the emotional responses brought on by the COVID‐19 pandemic. Psychiatric manifestations may be more important than the physical symptoms of TSC. Specific attention and close follow‐up to these vulnerable subjects during the period of pandemic are primordial to reduce the risk of developing chronic psychotic disorders.

## CONFLICTS OF INTEREST

All authors declare that they have no conflicts of interest to disclose.

## AUTHOR CONTRIBUTIONS

LB and HBA conceived the ideas and led the writing. HG, MS, and EK involved in writing. LM did the editing.

## ETHICAL APPROVAL

An informed consent publication was obtained from the patient and his father.

## CONSENT

Published with written consent of the patient.

## Data Availability

The data that support the findings of this study are available from the corresponding author upon reasonable request.
